# Morphological structures and histochemistry of roots and shoots in *Myricaria laxiflora* (Tamaricaceae)

**DOI:** 10.1515/biol-2021-0049

**Published:** 2021-05-10

**Authors:** Linbao Li, Di Wu, Qiaoling Zhen, Jun Zhang, Liwen Qiu, Guiyun Huang, Chaodong Yang

**Affiliations:** Rare Plants Research Institute of Yangtze River, China Three Gorges Corporation, Yichang, Hubei 443000, China; Engineering Research Center of Ecology and Agriculture Use of Wetland, Ministry of Education, Hubei Key Laboratory of Waterlogging Disaster and Agricultural Use of Wetland, Yangtze University, Jingzhou, Hubei 434025, China

**Keywords:** aerenchyma, apoplastic barriers, endodermis, lignified cortex and hypodermis, thick papillose cuticle

## Abstract

*Myricaria laxiflora* (Tamaricaceae) is an endangered plant that is narrowly distributed in the riparian zone of the Three Gorges, along the Yangtze River, China. Using bright-field and epifluorescence microscopy, we investigated the anatomical and histochemical features that allow this species to tolerate both submerged and terrestrial environments. The adventitious roots of *Myr. laxiflora* had an endodermis with Casparian bands and suberin lamellae; the cortex and hypodermal walls had lignified thickenings in the primary structure. In the mature roots, the secondary structure had cork. The apoplastic barriers in stems consisted of a lignified fiber ring and a cuticle at the young stage and cork at the mature stage. The leaves had two layers of palisade tissue, a hyaline epidermis, sunken stomata, and a thick, papillose cuticle. Aerenchyma presented in the roots and shoots. Several *Myr. laxiflora* structures, including aerenchyma, apoplastic barriers in the roots and shoots, were adapted to riparian habitats. In addition, shoots had typical xerophyte features, including small leaves, bilayer palisade tissues, sunken stomata, a thick, papillose cuticle, and a hyaline epidermis. Thus, our study identified several anatomical features that may permit *Myr. laxiflora* to thrive in the riparian zone of the Three Gorges, China.

## 1 Introduction

*Myricaria laxiflora* (Tamaricaceae) is an endangered species that prior to the construction of the Three Gorges Dam (TGD) was narrowly distributed in the riparian zone along the Yangtze River, China, from Banan County, Chongqing Province, to Zhijiang County, Hubei Province [1,2,3,4,5]. After TGD construction was completed in 2009, only a few natural populations of *Myr. laxiflora* remained, all downstream of Yidu and Zhijiang counties; as *Myr. laxiflora* habitats upstream of the TGD were lost, some of the plants from the upstream localities have been preserved *ex situ* [4,6,7,8,9]. In its native environment along the Yangtze River, *Myr. laxiflora* remains dormant while completely submerged during summer flood pulses and then sprouts in the autumn and winter after the floods recede [1,2,3,4,5]. *Myr. laxiflora* may represent a promising plant with which to restore the ecology of Yangtze River after the degradation associated with TGD construction [8,9,10,11,12,13,14].

Like many other wetland plants, *Myr. laxiflora* is typically subjected to anoxic submersion during summer flooding [2,3,15,16,17]. Aquatic and amphibious plants have aerenchyma and tight barriers to store and retain oxygen in anoxic conditions and during water–solute exchanges [18,19,20,21]. In the amphibious species *Cynodon dactylon*, *Artemisia lavandulaefolia*, and *Alternanthera philoxeroides*, which we have studied from the Jianghan Plain down to the Three Gorges, air spaces included aerenchyma and pith cavities in roots and shoots, and barriers included the endodermis, exodermis, and suberized peripheral ring [22,23,24,25]. *Myr. laxiflora* growing in the riparian zone of the Yangtze River may have aerenchyma and structures similar to these amphibious species.

Other species in the Tamaricaceae that are closely related to *Myr. laxiflora* have diverse habitats and are widely distributed in mountainous, cold, and arid regions worldwide as well as in those with saline-alkali soils. Plants in the Tamaricaceae are often used for ecological restoration [26,27,28,29,30,31,32,33]. In this family, xerophyte shoots have abundant palisade tissues under the epidermis [34,35,36,37,38,39,40,41]; the epidermis itself has a thick, papillose cuticle [28,36,42] and sunken stomata [38,43]. In addition, species that belong to Tamaricaceae have deep roots [32,34,44,45,46], which represent an adaptation to drought stress [47,48,49].

The structure and physiology of *Myr. laxiflora* seeds and shoots may play important roles in the propagation of this species as well as its invasion of new habitats [8,9,10,11,12,13,14,29,50]. However, little is known of the anatomical and histochemical features that allow *Myr. laxiflora* to tolerate submersion and exposure. To our knowledge, the only relevant previous study of this species showed that the surfaces of young branches had smooth, thin cuticles [29].

To address this knowledge gap, we aimed to investigate whether the anatomical and histochemical features of *Myr. laxiflora* were consistent with its tolerance to submersion as well as to diverse terrestrial environments. Evidence of such adaptative features might help to explain the ability of this plant to thrive despite summer dormancy and to grow in diverse terrestrial environments during the spring, autumn, and winter. To study the structures of roots and shoots, we analyzed the anatomical and histochemical characters of *Myr. laxiflora* samples, primarily using berberine hemisulfate–aniline blue (BAB) to visualize Casparian bands and lignified walls, Sudan red 7B (SR7B) to visualize suberin lamellae, and toluidine blue O (TBO) to visualize anatomical features.

## 2 Materials and methods

### 2.1 Sample collection and processing

In October 2019, we collected adventitious roots, stems, and leaves specimens of *Myr. laxiflora* at the riparian of the Yangtze River in Yidu County, Hubei, China. Approximately 50 adventitious roots and 20 shoots with leaves were collected from 10 individuals.

Adventitious root and leaf samples were fixed in formaldehyde–alcohol–acetic acid immediately following collection [51]. After fixation, the root tissues were sectioned freehand under a stereoscope (JNOEC JSZ6, China), using a two-sided blade razor. Adventitious root samples (∼30–80 mm long) were sectioned at 10, 20, 30, 40, or 50 mm from the root tip. Aged tissue with attached cortex was sloughed off. Each distance from the root tip was represented by 3–6 sections from different samples per stain.

Shoot bases were immersed in tap water immediately following collection. Shoots (∼150–270 mm long) were sectioned at 10, 20, 30, 40, and 50 mm from the shoot apex. Each distance from the shoot base was represented by 3–6 sections from different samples per stain. Sections (10–30 µm thick) were cut in the middle of the seedling leaves. Leaves were represented by 3–6 sections from different samples per stain.

### 2.2 Histochemistry and microscopy

Sections were stained with one of three stains. SR7B was used to identify suberin in the cell walls [52], BAB was used to identify Casparian bands and lignin in the cell walls [53,54], and TBO was used to visualize tissue structures [55,56]. All specimens were examined using bright-field microscopy under a Leica DME microscope and photographed with a digital camera (Nikon E5400, Japan). Specimens stained with BAB were viewed under an Olympus IX71 epifluorescence microscope and photographed with a digital camera (RZ200C–21, China) [22].

## 3 Results and discussion

### 3.1 General structure

**Figure 1:**
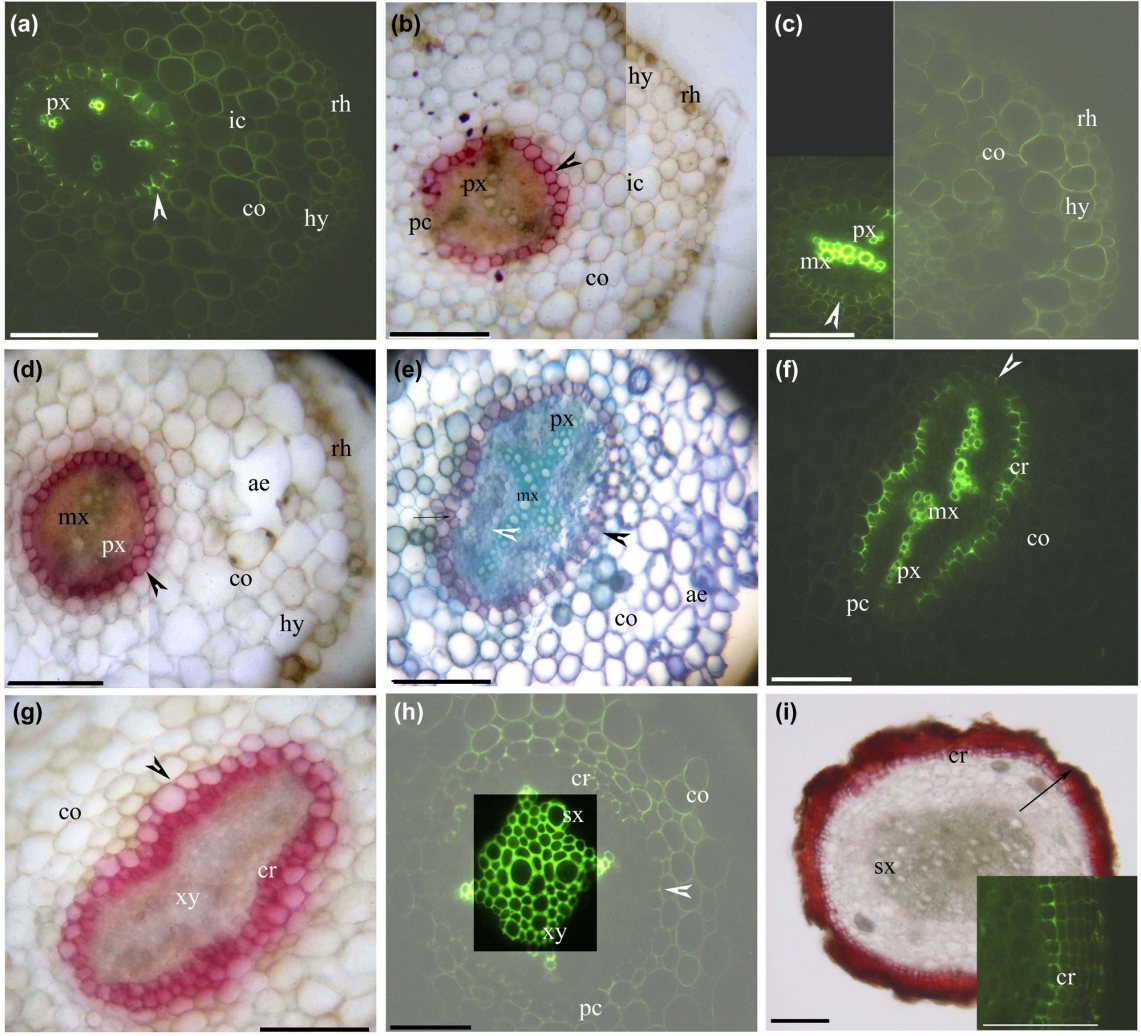
Photomicrographs of thick adventitious roots (50–80 mm long) of *Myricaria laxiflora*, showing some of the secondary growth; scale bars = 50 µm. (a) Protoxylem, endodermis (arrowhead), lignified cortex, intercellular space, hypodermis, and rhizodermis. Staining: BAB. (b) Protoxylem, endodermis (arrowhead), passage cells, cortex, intercellular space, hypodermis, and rhizodermis. Staining: SR7B. (c) Protoxylem, metaxylem, endodermis (arrowhead), lignified cortex, hypodermis, and rhizodermis. Staining: BAB. (d) Protoxylem, metaxylem, endodermis (arrowhead), cortex, aerenchyma, hypodermis, and rhizodermis. Staining: SR7B. (e) Protoxylem, vascular cambia (white arrowhead), divided pericycle (arrow), endodermis (black arrowhead), cortex, and aerenchyma. Staining: TBO. (f) Protoxylem, metaxylem, cork, endodermis (arrowhead), passage cells, and cortex. Staining: BAB. (g) Primary xylem, cork, endodermis (arrowhead), and cortex. Staining: SR7B. (h) Primary xylem, secondary xylem, cork, endodermis (arrowhead), passage cells, and lignified cortex. Staining: BAB. (i) Secondary xylem, cork, and bark (whole arrow). Staining: SR7B. Inset shows cork. Staining: BAB. Abbreviations used in the figure are as follows: ae – aerenchyma; BAB – berberine sulfate–aniline blue; ch – chloroplast; cr – cork; co – cortex; cu – cuticle; ep – epidermis; hy – hypodermis; ic – intercellular space; mx – metaxylem; pc – passage cells; pt – palisade tissue; f – phloem fibers; pi – pith; xy – primary xylem; px – protoxylem; rh – rhizodermis; st – spongy tissue; SR7B – Sudan red 7B; sx – secondary xylem; TBO – toluidine blue O; ve – vein.

**Figure 2:**
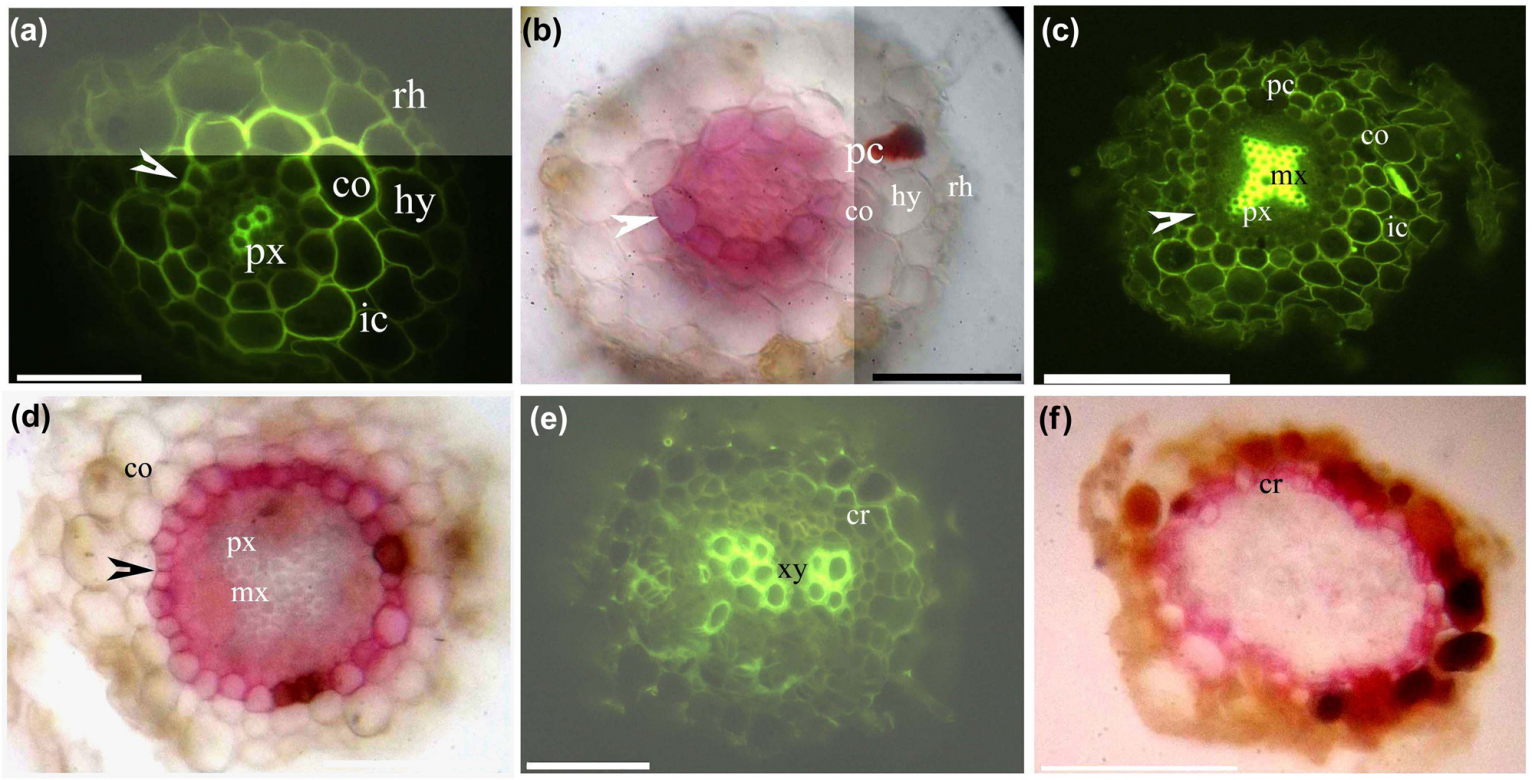
Photomicrographs of *Myricaria laxiflora* fine adventitious roots (30–50 mm long); scale bars = 50 µm. (a) Protoxylem, endodermis (arrowhead), lignified cortex, intercellular space, hypodermis, and rhizodermis. Staining: BAB. (b) Endodermis (arrowhead), passage cells, cortex, hypodermis, and rhizodermis. Staining: SR7B. (c) Protoxylem, metaxylem, endodermis (arrowhead), passage cells, lignified cortex, intercellular space. Staining: BAB. (d) Protoxylem, metaxylem, endodermis (arrowhead), cortex. Staining: SR7B. (e) Primary xylem, cork. Staining: BAB. (f) Cork. Staining: SR7B.

**Figure 3:**
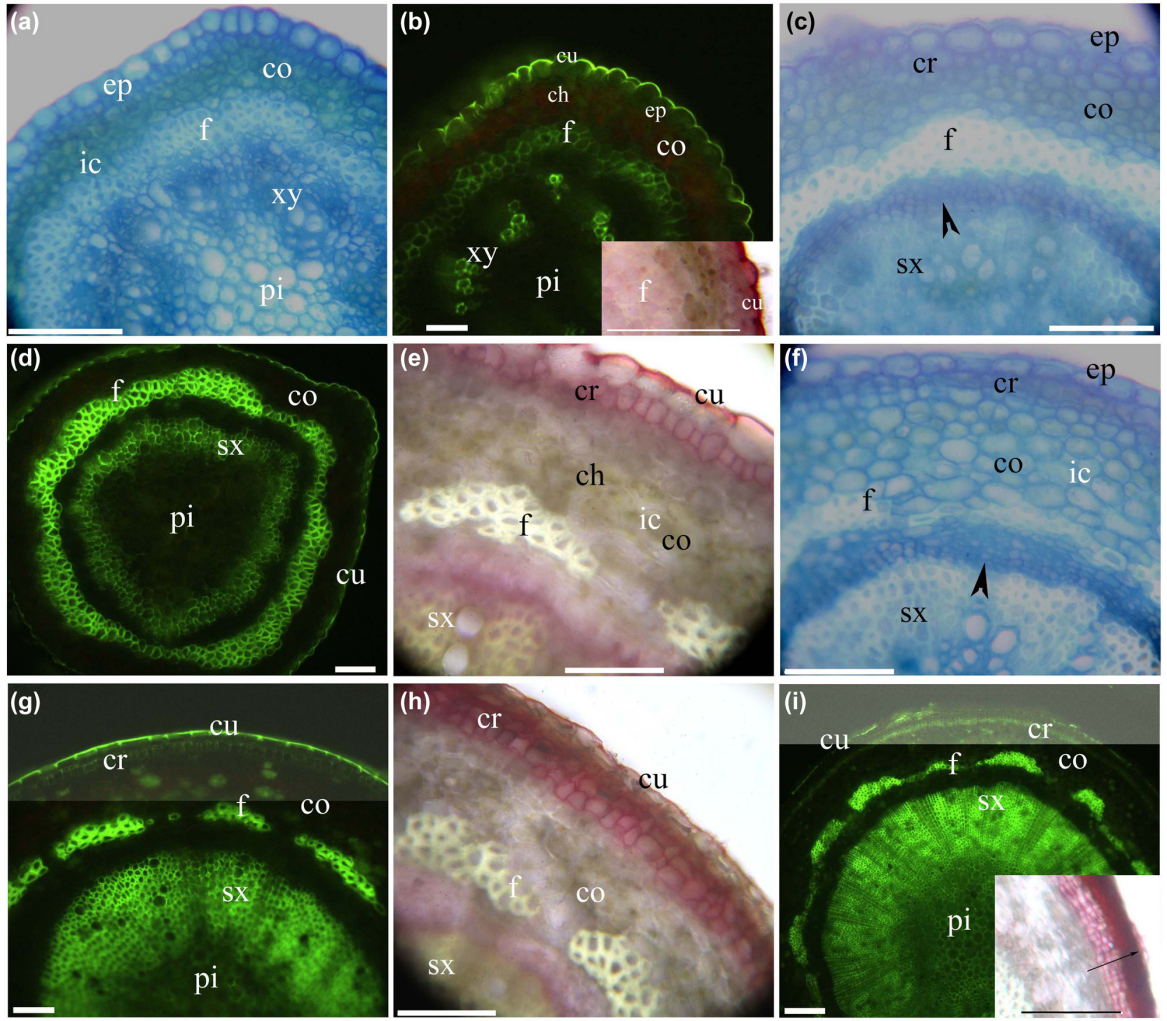
Photomicrographs of *Myricaria laxiflora* stems (150–270 mm long). Scale bars = 50 µm. (a) Pith, primary xylem, phloem fibers, cortex, intercellular space, and epidermis. Staining: TBO. (b) Pith, primary xylem, phloem fibers, cortex, chloroplast, epidermis, and cuticle. Staining: BAB. Inset shows phloem fibers and cuticle. Staining: SR7B. (c) Secondary xylem, vascular cambia (arrowhead), phloem fibers, cortex, divided cork, and epidermis. Staining: TBO. (d) Pith, secondary xylem, phloem fibers, cortex, and cuticle. Staining: BAB. (e) Secondary xylem, phloem fibers, cortex, intercellular space, chloroplast, cork, and cuticle. Staining: SR7B. (f) Secondary xylem, vascular cambia (arrowhead), phloem fibers, cortex, intercellular space, cork, and epidermis. Staining: TBO. (g) Pith, secondary xylem, phloem fibers, cortex, cork, and cuticle. Staining: BAB. (h) Secondary xylem, phloem fibers, cortex, cork, and cuticle. Staining: SR7B. (i) Pith, secondary xylem, phloem fibers, cortex, cork, and cuticle. Staining: BAB. Inset shows bark (arrow). Staining: SR7B.

**Figure 4:**
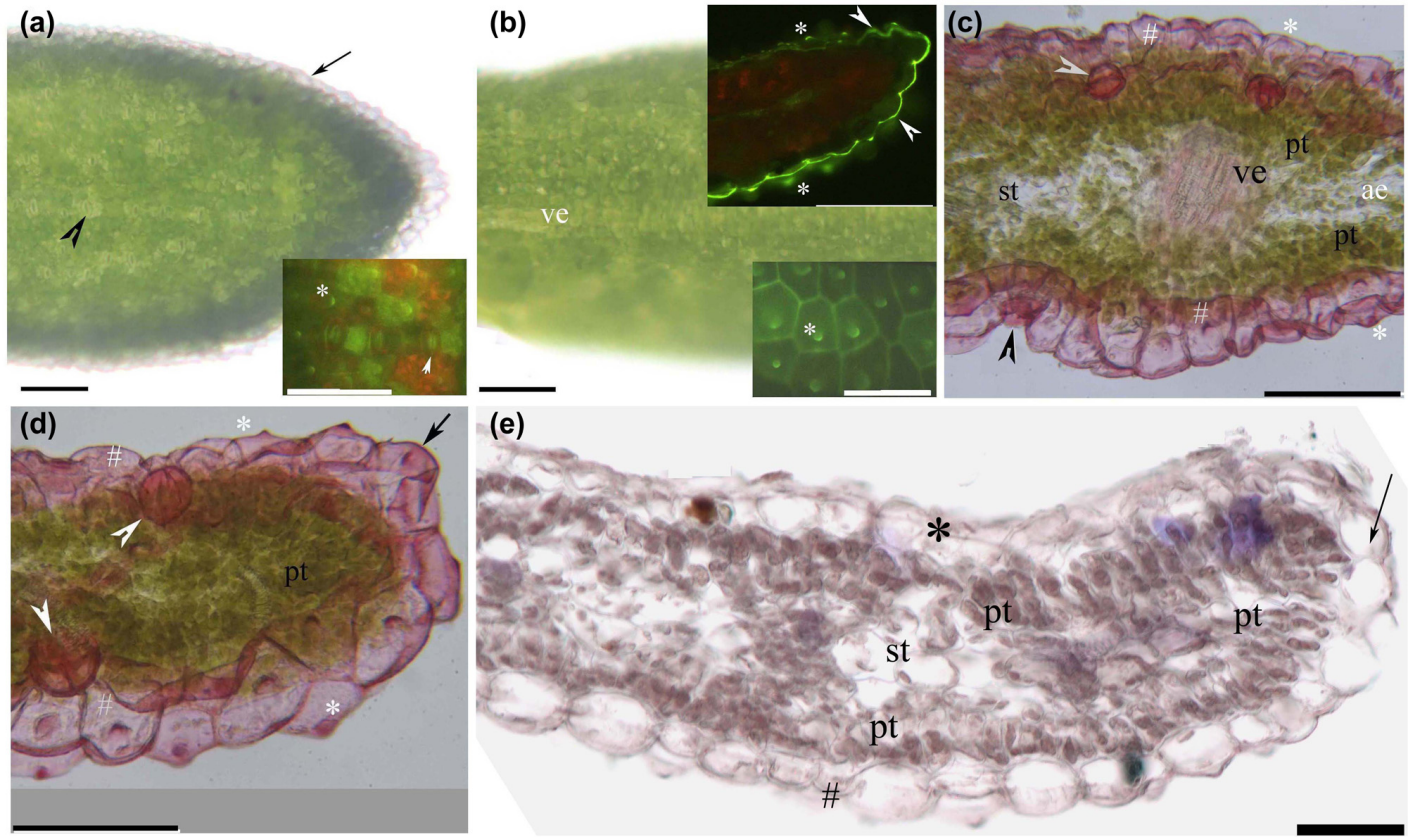
Photomicrographs of *Myricaria laxiflora* leaves. Scale bar = 50 µm. (a) Adaxial surface, stomata (arrowhead), and hyaline epidermal margin (arrow). Unstained. Inset shows stomata (arrowhead), and fine papillae (*). Staining: BAB. (b) Abaxial surface and vein. Unstained. Upper inset shows lower and marginal epidermal walls with thick cuticle (arrowhead) and papillae (*). Staining: BAB. Lower inset shows large papillae (*). Staining: BAB. (c) Middle blade, vein, upper epidermis with cuticle and fine papillae (white #), lower epidermis with cuticle and large papillae (gray #), stomata (arrowhead), palisade tissue, aerenchyma, spongy tissue, and papillae (*). Staining: SR7B. (d) Marginal blade, upper epidermis with cuticle and fine papillae (white #), lower epidermis with cuticle and large papillae (gray #), stomata (arrowhead), palisade tissue, papillae (*), and marginal epidermis (arrow). Staining: SR7B. (e) Blade, upper epidermis (*), lower epidermis (#), palisade tissue, spongy tissue, and hyaline epidermal margin (arrow). Staining: TBO.

*Myr. laxiflora* had thick adventitious roots (Figure 1), fine adventitious roots (Figure 2), and shoots (Figures 3 and 4). The thick adventitious roots possessed four to five layers of cortex cells in the primary structure (Figure 1a–d); in the secondary structure, the cortex sloughed off with the bark (Figure 1e–i). The fine adventitious roots contained one or two layers of cortex cells in the primary structure and only cork in the secondary structure (Figure 2). Both thick and thin adventitious roots had diarch to tetrarch stele with differentiated proto- and metaxylem, a cortex with an endodermis, a hypodermis, and a rhizodermis. The cortex and hypodermal walls had lignified thickenings. Aerenchyma were present in the root cortices.

The *Myr. laxiflora* stem possessed cork and an epidermis as well as a lignified phloem fiber ring enclosing a central cylinder of bundles internal to the cortex (Figure 3a–i). The *Myr. laxiflora* leaf had palisade tissue, a hyaline marginal epidermis, stomata, and a cuticle with a papillose surface (Figure 4a–f).

### 3.2 Structure of the thick adventitious root

At 10 mm from the root tip, the stele had diarch and tetrarch protoxylem poles, the endodermis had Casparian bands and almost complete suberin lamellae (only a few passage cells), the cortex and hypodermal walls had lignified thickenings, and an intercellular space appeared within the cortex (Figure 1a and b). At 20 mm from the root tip, the stele had metaxylem poles, the endodermis had almost complete suberin lamellae, the cortex had irregular lysigenous aerenchyma, and the rhizodermis was still intact (Figure 1c and d). At 30 mm from the root tip, a redivided pericycle also formed phellogen to begin cork production and the endodermis had few passage cells (Figure 1e–g). At about 40 mm from the root tip, the pericycle over the protoxylem poles and the cells between the primary xylem and the primary phloem had become a vascular cambium to initiate the secondary xylem, the cork was partially undeveloped, and the cortex begin to slough off (Figure 1e–h). At >50 mm from the root tip (mature adventitious roots), the cortex and hypodermis had been sloughed off, the stele has a secondary xylem, and the cork had suberized to form bark (Figure 1i).

### 3.3 Structure of the fine adventitious root

At 10 mm from the root tip, Casparian bands, suberin lamellae, and passage cells were present on the endodermis, the cortex and hypodermal walls had lignified thickenings, and the stele had a diarch protoxylem (Figure 2a and b). At 20 mm from the root tip, the endodermis had almost complete suberin lamellae with a few passage cells, the stele had a metaxylem, and the cortex and hypodermis begin to slough off (Figure 2c and d). At 30 mm from the root tip, the pericycle redivided to form phellogen and produce suberized cork, while the stele had only primary xylem (Figure 2e and f).

We demonstrated that the primary structures of the thick and fine adventitious roots exhibit similar anatomical and histochemical features of *Myr. laxiflora*. *Myr. laxiflora* roots had a suberized endodermis and a lignified hypodermis, while the cortex and hypodermal walls had lignified thickenings near the endodermis. The cortex of the thick adventitious roots had more cell layers than that of the fine adventitious roots. In addition, the thick adventitious roots had a secondary structure containing cork, as commonly observed in eudicots [57,58,59]. In contrast, the fine adventitious roots had only primary xylem.

The young roots of *Myr. laxiflora* were similar in structure to the young roots of *Oenanthe javanica* and *Alt. philoxeroides* [25,60]. However, the hypodermis of *O. javanica* has more cell layers than that of *Myr. laxiflora* as well as a cortex with spacious aerenchyma (although this cortex lacks lignified walls); in addition, unlike *Myr. laxiflora*, the roots of *O. javanica* are surrounded by aerenchyma, and the walls possess suberin lamellae [60]. The cortices and hypodermis of the aquatic roots of *Alt. philoxeroides* have lignified walls and aerenchyma [25], as well as broccoli and *Cardamine hupingshanensis* [61,62,63]. It is possible that the lignified thickenings we observed in the roots of *Myr. laxiflora* relate to the riparian habitats of the Three Gorges [25,61,62,63].

The roots of wetland or aquatic eudicots from Jianghan Plain (China) or from the Amazon Basin, such as *Art. lavandulaefolia*, *Art. selengensis*, *Ranunculus trichophyllus*, and *Tabernaemontana juruana*, possess an endodermis, a uniseriate exodermis, and a cortex that lacks lignified walls [24,64,65]. In contrast, the roots of wetland grasses, such as *Oryza sativa*, *Phalaris arundinacea*, *Phragmites australis*, and *Zizania latifolia*, possess an endodermis and a multiseriate exodermis [20,22,23,66,67]. The barriers of these wetland or aquatic species were stronger than *Myr. laxiflora* with an endodermis and lignified cortex as well as hypodermis in roots.

### 3.4 Stem structure

The stem had a lignified phloem fiber ring, enclosing a central cylinder of bundles internal to the cortex, and an epidermis with a thick cuticle. At 10 mm from the new shoot apex, the fiber ring enclosed vascular bundles, and a spacious pith was present in the center (Figure 3a and b). At 30–40 mm from the new shoot apex, the vascular cambium produced an internal secondary xylem; the phloem fibers had strengthened and lignified; and the cortical cells had redivided to form suberized cork, one cell layer thick, under the epidermis (Figure 3c–e). At the new shoot base, the cork had several layers of suberized cells (Figure 3f–h). In 1-year-old shoots, the cylinder bundles had spacious secondary xylem, and the cork has suberized to form bark (Figure 3i). Intercellular spaces and chloroplasts were present in the stem cortices (Figure 3a, b, e, and f).

Young stems of *Myr. laxiflora* possessed a lignified fiber ring, a thick cuticle, and a cortex either with chloroplasts and small aerenchyma or with one layer of cork cells. In contrast, mature stems had prominent secondary xylem in the center of the stem and a thick bark. In contrast, Zhang et al. [29] found that young branches of *Myr. laxiflora* had smooth, thin cuticles. The lignified fiber ring in the young stems of *Myr. laxiflora* was similar to the lignified sclerenchymal ring observed in *C. hupingshanensis*, *P. arundinacea*, and *Z. latifolia* [22,23,63,67]. This ring might serve to increase the mechanical strength of the young stem. Suberized cork, which is commonly observed in eudicots [58,59], is similar to the phellem of *Art. selengensis* and *Alt. philoxeroides* [24,25] and to the suberized and lignified peripheral mechanical ring in the *Paspalum distichum*, *Pha. arundinacea*, and *Z. latifolia* [22,23,67]. This suggests that *Myr. laxiflora’s* tolerance to flooding is typical of amphibious plants. The shoot cortices have chloroplasts, which are present in other Tamaricaceae species [28,30,68]. These observations indicate that *Myr. laxiflora* has features that belong to Tamaricaceae taxology and serve as adaptations to the environments in the Three Gorges.

### 3.5 Leaf structure

The upper surface of the leaf has obvious stomata, small epidermal cells, a thin cuticle, and fine papillae. The lower surface and edge of the leaf have sunken stomata, large epidermal cells, a thick cuticle, and large papillae (Figure 4a–d, e). The edges of the epidermal cells are hyaline (Figure 4a and e). Palisade tissue was observed below and above the adaxial and abaxial epidermis, respectively; scant spongy mesophyll tissue was observed between the layers of palisade tissue (Figure 4c–e). Aerenchyma was present in the middle of the leaf blade (Figure 4c).

The leaves of *Myr. laxiflora* are typical of xerophytes adapted to arid environments: they are small and have two layers of palisade tissue, sunken stomata, and a thick papillose cuticle. Bilayer palisade tissues are also found in several other xerophytes, including *Myr. bracteate* [26], *Myr. germanica* [70], *Reaumuria soongoriea* [35], *Tamarix ramosissima* [30], *Elaeagnus angustifolia* [36], *Eschweilera tenuifolia* [69], *Populus euphratica* [36,40], *Peganum nigellastrum* [39], *Alhagi sparsifolia* [34], and *Ziziphus jujuba* var. *spinosa* [41]. Similarly, the leaves of several xerophyte plants, including *Myr. germanica* [70], *Tam. laxa* [28,42], *Tam. ramosissima* [30], *Tam. chinensis* [68], *Ela. angustifolia* [36], and *Caragana* spp. [37,38], have sunken stomata, thick cuticles, and surface papillae [59]. The epidermis at the abaxial margins of the leaves of *Myr. laxiflora* was largely hyaline and may function similar to the white hairs on xerophyte leaves [36,37] or the hyaline tips of bryophyte leaves [71].

In plant tissues, aerenchyma help to retain oxygen when the plant is submerged, in order to improve survival [15,16,17,20,72,73]. The roots of *Myr. laxiflora* had aerenchyma and histochemical features similar to those of *Alt. philoxeroides* aquatic roots, even though *Alt. philoxeroides* shoots have large air spaces [25], and the leaves have lysigenous of xeromorphic New Zealand hemp [59], while *Myr. laxiflora* shoots have narrow intercellular spaces. In contrast, the shoots of wetland plants, such as *Pas. distichum*, *Art. lavandulaefolia*, and *Art. selengensis*, have spacious pith cavities and cortical lacunae, which might facilitate survival when submerged over long periods [22,23,24,64,67].

## 4 Conclusion

We identified that *Myr. laxiflora* have typical amphibious plant features, including apoplastic barriers consisting of the endodermis, lignified wall thickenings, cork, and cuticle as well as the aerenchyma, suggesting that *Myr. laxiflora* is well adapted to the riparian habitats of the Three Gorges along the Yangtze River [16,17,18,19,24,25,54,60,63,72]. The shoots of *Myr. laxiflora* have typical xerophyte features, common across the Tamaricaceae, including small leaves, bilayer palisade tissues, sunken stomata, a thick papillose cuticle, and a largely hyaline epidermis [26,28,30,35,36,37,40,42,59,68,69]. Our results help to explain how the rare plant *Myr. laxiflora* survives in flooded and receded environments and may help to contextualize the taxonomy, evolution, and phylogeny of *Myr. laxiflora* within Tamaricaceae.
